# Mental health and psychological distress in healthcare-related students post-pandemic: a cross-sectional study

**DOI:** 10.3389/fpsyg.2026.1801247

**Published:** 2026-07-20

**Authors:** Rui Guo, Zhiyong Yuan, Wei Yan, Zhenjiang Huang, Rong Rong, Yusheng Liu, Yiran ZhouGuo, Mannan Abdul, Yan Li, Guoqing Feng, Ziyang Zhang, Qing Zhou, Yanan Wang

**Affiliations:** 1 Pediatric Teaching and Research Office, Heze Medical College, Heze, China; 2 Heze Medical College, Heze, China; 3 Genetics Teaching and Research Office, Xuzhou Medical University, Xuzhou, China; 4 College of Medical Technology, Xuzhou Medical University, Xuzhou, China; 5 Foreign Languages Department, Xuzhou Medical University, Xuzhou, Jiangsu, China; 6 Department of Anesthesiology, EENT Hospital of Fudan University, Shanghai, China; 7 China Academy of Traditional Chinese Medicine, Jining Hospital, Xiyuan Hospital, Jining, China; 8 Department of Nuclear Medicine, The Affiliated Hospital of Xuzhou Medical University, Xuzhou, China; 9 Xuzhou Yiyang Education Consulting Company Limited, Xuzhou, Jiangsu, China

**Keywords:** anxiety, COVID-19, depression, health related students, mental health

## Abstract

**Background:**

University students, particularly those in healthcare-related fields, face substantial mental health challenges exacerbated by the Coronavirus disease 2019 (COVID-19) pandemic. Despite the recognized importance of mental health in mitigating psychological distress, research assessing mental health related depression and anxiety among these healthcare-related students remains limited.

**Methods:**

A cross-sectional study was conducted between April and May 2023 at a medical university in Shandong Province, China. A total of 10,267 students completed the Symptom Checklist-90 (SCL-90), 12,950 completed the Self-Rating Depression Scale (SDS), and 12,735 completed the Self-Rating Anxiety Scale (SAS). Data were analyzed using descriptive statistics, *t*-tests, ANOVA, and logistic regression to assess the prevalence and risk factors for depression and anxiety.

**Results:**

Among 12,950 medical university students, 67.49% were asymptomatic for depression, while 15.89, 16.10, and 0.52% exhibited mild, moderate, and severe depressive symptoms, respectively. Anxiety assessment of 12,735 students showed 89.93% were asymptomatic, with mild, moderate, and severe anxiety present in 8.04, 1.56, and 0.46%. SDS scores differed significantly across academic grades (*p* < 0.001) but not by gender, whereas females had slightly higher SAS scores than males (*p* = 0.015). The prevalence of depression and anxiety varied significantly across academic departments (*p* < 0.001). Multivariable analysis identified academic grade as an independent predictor of both depression and anxiety severity, with department and gender additionally associated with moderate depression.

**Conclusion:**

Healthcare related students demonstrate moderate levels of psychological distress, with distinct demographic and academic patterns. Targeted interventions are urgently needed, particularly for younger students and those in high-stress majors. Enhancing mental health could play a crucial role in mitigating the burden of depression and anxiety in this vulnerable population.

## Introduction

The mental health of university students has become a global concern, particularly in the wake of the COVID-19 pandemic, which has exacerbated existing challenges (Korhonen et al., 2025; Xiong et al., 2020; Zarowski et al., 2024). Healthcare related-students, due to the rigor and demands of their education, are particularly vulnerable to psychological distress (Nair et al., 2023). Depression and anxiety are among the most common mental health issues affecting this population, with significant implications for their academic performance, professional development, and long-term wellbeing (Quek et al., 2019). Mental health literacy (MHL), defined as the knowledge and beliefs about mental disorders that aid in their recognition, management, or prevention, plays a critical role in promoting mental health and reducing the stigma associated with mental illness (Gorczynski and Sims-Schouten, 2024). However, despite its importance, there is a lack of research assessing MHL and its association with mental health outcomes among healthcare related students (Jabari et al., 2025). Several factors contribute to the psychological burden faced by healthcare-related students, including academic pressure, clinical responsibilities, social isolation, and the transition to adulthood (Bergmann et al., 2019; Shiraly et al., 2024). The COVID-19 pandemic has introduced additional stressors such as uncertainty about the future, disruptions to education, and concerns about personal and family health (Nikopoulou et al., 2022; Son et al., 2020; Weierstall-Pust et al., 2022). These stressors have collectively heightened the risk of developing mental health issues among students. Existing studies suggest that younger students are particularly susceptible to depression and anxiety (Mofatteh, 2020; Varma et al., 2021). Moreover, certain academic disciplines, notably those involving intensive clinical exposure and demanding curricula, such as Traditional Chinese Medicine, Pharmacy, and Nursing, may predispose students to higher levels of stress and psychological morbidity (Li et al., 2023; Mohamed et al., 2024). Given the potential long-term impact of mental health issues on healthcare-related students and the healthcare system, it is imperative to understand the prevalence and determinants of psychological distress in this group. This study aims to fill the gap by investigating mental health status, including depression and anxiety, among healthcare-related students.

## Materials and methods

### Study design and setting

A cross-sectional survey was conducted between April and May 2023 at a Heze Medical College in Heze, Shandong Province, China. The study protocol was reviewed and approved by the Ethics Committee of Heze Medical College (Approval No. 86). Participation was voluntary, and the confidentiality of all responses was ensured. The ethics committee/institutional review board waived the requirement for written informed consent for participation because the study involved anonymized survey data and posed minimal risk to participants.

### Participants and data collection

Participants were full-time undergraduate students enrolled at the university medical college. Departments represented broader academic categories, while majors referred to specific professional training programs within those departments. Inclusion criteria required students to be aged 15 years or older, enrolled in one of the college’s program and capable of understanding and completing the survey independently. Students who completed the survey in less than 300 s were excluded to ensure data quality. Cluster sampling was employed based on grade, department, and major. Surveys were administered electronically, and informed consent was obtained from all participants prior to data collection. A self-designed questionnaire collected data on participants’ age, gender, grade, department, and major.

### Measurement scales

(1) Symptom Checklist-90 (SCL-90): A 90-item scale measuring a range of psychological symptoms across nine domains, including depression, anxiety, and obsessive-compulsive tendencies. Scores were interpreted based on national norms (Du et al., 2022).
(2) Self-Rating Depression Scale (SDS): A 20-item instrument assessing depressive symptoms. Scores were categorized into mild, moderate, and severe depression based on established cut-offs (Shen et al., 2012).
(3) Self-Rating Anxiety Scale (SAS): A 20-item instrument evaluating anxiety symptoms. Scores were categorized into mild, moderate, and severe anxiety (Shen et al., 2012).

### Statistical analysis

Data were analyzed using SPSS version 25.0. Descriptive statistics were used to summarize demographic and mental health characteristics. Group differences were assessed using *t*-tests and one-way ANOVA. Logistic regression analyses were performed to identify factors associated with depression and anxiety. The coding scheme for all variables included in the binary logistic regression models was presented in Supplementary Table 1.

## Results

### Prevalence of depressive and anxiety symptoms among medical university students

Among the 12,950 medical university students assessed for depressive symptoms, 8,740 (67.49%) were classified as asymptomatic. Mild and moderate depressive symptoms were observed in 2,058 (15.89%) and 2,085 (16.10%) students, respectively, while severe depression was identified in 67 students (0.52%). Assessment of anxiety symptoms was completed for 12,735 students. The majority of participants, 11,453 (89.93%), were asymptomatic. Mild anxiety was reported in 1,024 students (8.04%), whereas moderate and severe anxiety were present in 199 (1.56%) and 59 (0.46%) students, respectively (Table 1).

**TABLE 1:** Prevalence of depression and anxiety among healthcare-related students.

	Depression (*n* = 12,950)				Anxiety (*n* = 12,735)			
	Asymptomatic	Mild	Moderate	Severe	Asymptomatic	Mild	Moderate	Severe
(*n*)	8,740	2,058	2,085	67	11,453	1,024	199	59
(%)	67.49	15.89	16.1	0.52	89.93%	8.04%	1.56%	0.46%

### SDS and SAS scores by gender and academic year

The mean SDS score was 45.94 ± 11.50 among male students and 46.34 ± 12.65 among female students, with no statistically significant difference between genders (*t* = 1.652, *p* = 0.099). In contrast, SDS scores had a statistical significance across academic years (F = 92.074, *p* < 0.001), with mean scores of 46.98 ± 11.50 in Grade 1, 46.92 ± 12.31 in Grade 2, and 43.87 ± 11.55 in Grade 3. For anxiety, the mean SAS score was 38.97 ± 8.63 in males and 39.39 ± 8.72 in females, showing a statistically significant gender difference (*t* = 2.431, *p* = 0.015). Significant differences in SAS scores were also observed across grades (F = 100.413, *p* < 0.001), with mean scores of 39.74 ± 8.82 in Grade 1, 39.86 ± 8.75 in Grade 2, and 37.42 ± 8.09 in Grade 3 (Table 2). Detailed detection rates of depression and anxiety according to gender and academic grade are presented in Supplementary Table 2.

**TABLE 2:** SDS and SAS scores by gender and academic year among healthcare-related students.

		SDS score (*n* = 12,950)					SAS score (*n* = 12,735)		
Gender		Academic year			Gender		Academic year		
Male	Female	Grade 1	Grade 2	Grade 3	Male	Female	Grade 1	Grade 2	Grade 3
45.94 ± 11.50	46.34 ± 12.65	46.98 ± 11.50	46.92 ± 12.31	43.87 ± 11.55	38.97 ± 8.63	39.39 ± 8.72	39.74 ± 8.82	39.86 ± 8.75	37.42 ± 8.09
*t* = 1.652		F = 92.074			*t* = 2.431		F = 100.413		
*p* = 0.099		*p* = 0.000**			*p* = 0.015*		*p* = 0.000**		

*p < 0.05,

**p < 0.001. Values are presented in mean ± SD.

### Prevalence of depression and anxiety across academic departments

The prevalence of depressive symptoms varied significantly across academic departments (χ^2^ = 318.757, *p* < 0.001). The proportion of asymptomatic students ranged from 61.27% in Nursing to 78.71% in Clinical Medicine, while moderate-to-severe depressive symptoms were more frequently observed in Nursing (21.73%) and Stomatology (20.52%) compared with other departments. Traditional Chinese Medicine and Health Management students showed similar distributions, with approximately one-third reporting at least mild depressive symptoms. In contrast, Clinical Medicine and Medical Technology students demonstrated higher proportions of asymptomatic status and lower rates of moderate and severe depression. Anxiety prevalence also differed significantly among departments (χ^2^ = 64.868, *p* < 0.001). The percentage of asymptomatic students exceeded 86% in all departments and was highest in Medical Technology (92.66%) and Stomatology (92.54%). Mild anxiety was most common in Traditional Chinese Medicine and Pharmacology students, whereas moderate and severe anxiety remained relatively uncommon across all departments, each accounting for less than 3% of cases (Table 3). Detailed SCL-90 factor scores, SDS scores, and SAS scores according to department and major are presented in Supplementary Tables 3–5.

**TABLE 3:** Prevalence of depression and anxiety among healthcare-related students across academic departments.

	Depression (*n* = 12,950)				Anxiety (*n* = 12,735)			
	Asymptomatic	Mild	Moderate	Severe	Asymptomatic	Mild	Moderate	Severe
TCM	981 (65.05)	268 (17.77)	245 (16.25)	14 (0.93)	1,292 (86.08)	159 (10.59)	39 (2.60)	11 (0.73)
Clinical Medicine	1,231 (78.71)	148 (9.46)	181 (11.57)	4 (0.26)	1,375 (91.36)	105 (6.98)	21 (1.40)	4 (0.27)
Health Management	1,011 (68.68)	262 (17.80)	194 (13.18)	5 (0.34)	1,282 (89.34)	121 (8.43)	23 (1.60)	9 (0.63)
Medical Technology	1,581 (74.47)	322 (15.17)	212 (9.99)	8 (0.38)	1,944 (92.66)	120 (5.72)	28 (1.33)	6 (0.29)
Stomatology	562 (66.27)	112 (13.21)	173 (20.40)	1 (0.12)	769 (92.54)	54 (6.50)	6 (0.72)	2 (0.24)
Nursing	2,822 (61.27)	783 (17.00)	975 (21.17)	26 (0.56)	4,039 (89.58)	377 (8.36)	70 (1.55)	23 (0.51)
Pharmacology	552 (66.59)	163 (19.66)	105 (12.67)	9 (1.09)	752 (87.85)	88 (10.28)	12 (1.40)	4 (0.47)
Total	8,740 (67.49)	2,058 (15.89)	2,085 (16.10)	67 (0.52)	11,453 (89.93)	1,024 (8.04)	199 (1.56)	59 (0.46)
χ2	318.757				64.868			
*p*	0.000**				0.000**			

**p < 0.001. Values are presented *n* (%).

### Multivariable analysis of factors associated with depression severity

Multinomial logistic regression analysis identified several factors associated with different levels of depressive symptoms among medical university students (Table 4). For mild depression, older age was associated with slightly lower odds of depressive symptoms (OR = 0.943, 95% CI: 0.895–0.993, *p* = 0.025), whereas higher academic grade (OR = 1.213, 95% CI: 1.122–1.312, *p* < 0.001) and department category (OR = 1.046, 95% CI: 1.017–1.076, *P* = 0.002) were associated with increased odds; gender and major were not significant predictors. In the moderate depression model, gender showed a significant association (OR = 1.709, 95% CI: 1.544–1.892, *p* < 0.001), along with academic grade (OR = 1.104, 95% CI: 1.027–1.187, *p* = 0.007) and department (OR = 1.128, 95% CI: 1.095–1.161, *p* < 0.001), while age and major were not significantly associated. No statistically significant associations were observed between age, gender, grade, department, or major and severe depression (all *p* > 0.05) (Table 4).

**TABLE 4:** Analysis of depression-related factors among healthcare-related students (*n* = 12,950).

	Parameters	Regression coefficient	z value	Wald χ^2^	*p*-value	Odd-ratio	95% CI
Mild	Age	−0.059	−2.242	5.025	0.025	0.943	0.895–0.993
	Gender	−0.016	−0.291	0.085	0.771	0.984	0.881–1.098
	Grade	0.193	4.827	23.297	0	1.213	1.122–1.312
	Department	0.045	3.13	9.797	0.002	1.046	1.017–1.076
	Major	0.003	0.674	0.454	0.5	1.003	0.995–1.010
	Intercept	−391.56	−4.814	23.17	0	0	0.000–0.000
Moderate	Age	−0.015	−0.702	0.493	0.483	0.985	0.944–1.028
	Gender	0.536	10.33	106.716	0	1.709	1.544–1.892
	Grade	0.099	2.684	7.204	0.007	1.104	1.027–1.187
	Department	0.12	8.004	64.069	0	1.128	1.095–1.161
	Major	−0.002	−0.502	0.252	0.616	0.998	0.990–1.006
	Intercept	−202.46	−2.707	7.326	0.007	0	0.000–0.000
Severe	Age	−0.065	−0.469	0.22	0.639	0.937	0.713–1.230
	Gender	−0.156	−0.539	0.291	0.59	0.855	0.485–1.509
	Grade	0.139	0.678	0.46	0.498	1.149	0.769–1.716
	Department	0.034	0.473	0.224	0.636	1.034	0.900–1.189
	Major	0.006	0.332	0.11	0.74	1.006	0.970–1.044
	Intercept	−284.3	−0.684	0.468	0.494	0	0.000–0.001

### Multivariable analysis of factors associated with anxiety severity

Multinomial logistic regression analysis examined factors associated with varying levels of anxiety among medical university students (Table 5). For mild anxiety, academic grade was significantly associated with increased odds (OR = 1.348, 95% CI: 1.221–1.489, *p* < 0.001), whereas age, gender, department, and major showed no statistically significant associations. In the moderate anxiety model, higher academic grade remained significantly associated with anxiety (OR = 1.564, 95% CI: 1.279–1.914, *p* < 0.001), while department was inversely associated with moderate anxiety (OR = 0.918, 95% CI: 0.847–0.995, *p* = 0.036); age, gender, and major were not significant predictors. For severe anxiety, academic grade was again significantly associated with increased odds (OR = 1.527, 95% CI: 1.069–2.181, *p* = 0.020), whereas age, gender, department, and major did not demonstrate statistically significant associations.

**TABLE 5:** Analysis of anxiety-related factors among healthcare-related students (*n* = 12,735).

	Parameter	Regression coefficient	z value	Wald χ^2^	*p*-value	Odd ratio	95% CI
Mild	Age	−0.016	−0.53	0.281	0.596	0.984	0.929–1.043
	Gender	0.076	1.067	1.138	0.286	1.079	0.938–1.242
	Grade	0.299	5.895	34.752	0	1.348	1.221–1.489
	Department	−0.019	−1.003	1.007	0.316	0.981	0.945–1.018
	Major	0	0.05	0.002	0.96	1	0.990–1.010
	Intercept	−605.81	−5.897	34.773	0	0	0.000–0.000
Moderate	Age	0.037	0.852	0.726	0.394	1.038	0.953–1.130
	Gender	−0.312	−1.84	3.387	0.066	0.732	0.525–1.020
	Grade	0.447	4.35	18.921	0	1.564	1.279–1.914
	Department	−0.086	−2.091	4.374	0.036	0.918	0.847–0.995
	Major	−0.005	−0.467	0.218	0.641	0.995	0.974–1.016
	Intercept	−908.7	−4.361	19.02	0	0	0.000–0.000
Severe	Age	0.058	0.824	0.679	0.41	1.06	0.923–1.218
	Gender	−0.111	−0.376	0.142	0.707	0.895	0.502–1.596
	Grade	0.423	2.329	5.422	0.02	1.527	1.069–2.181
	Department	−0.078	−1.086	1.179	0.278	0.925	0.804–1.065
	Major	0.022	1.19	1.415	0.234	1.022	0.986–1.059
	Intercept	−862.5	−2.342	5.485	0.019	0	0.000–0.000

## Discussion

This large-scale cross-sectional study offers a comprehensive and contemporary assessment of the mental health status among medical university students in China. The findings reveal a considerable prevalence of depressive and anxiety symptoms, underscoring a growing public health challenge. Disparities across gender, academic year, and academic departments suggest that psychological distress among healthcare-related students is shaped by a complex interplay of demographic and educational factors (Idowu et al., 2022; Roy et al., 2025). These findings align with and extend prior research highlighting the elevated psychological burden faced by healthcare-related students globally, particularly amid the evolving pressures in the post-pandemic era (Dong et al., 2024; Feyzbabaie et al., 2025; Li et al., 2025; Popescu et al., 2023).

### Prevalence of psychological distress

The observed prevalence of depressive symptoms (33.51%) and anxiety symptoms (10.07%) reflects a worrying trend among healthcare-related students. The predominance of mild and moderate severity indicates a critical window for early identification and intervention before the progression to more severe psychiatric morbidity. These data emphasize the urgent need for comprehensive screening programs embedded within academic institutions.

### Gender and academic year disparities

Consistent with global trends, female students exhibited significantly higher levels of depression and anxiety compared to their male counterparts (Al-Amin et al., 2025; Gao et al., 2020; Zhu et al., 2025). This gender disparity may be attributed to a combination of biological susceptibility, gender-specific stressors, and societal expectations. Notably, students in their first and second academic years reported heightened psychological distress (Nosè et al., 2025), likely reflecting transitional challenges, academic pressure, and limited coping resources during the early stages of their medical education.

### Impact of academic discipline

Marked disparities were also observed across academic disciplines, with students enrolled in Traditional Chinese Medicine, Pharmacy, and Nursing programs exhibiting the highest psychological distress. The intense clinical training, demanding coursework, and early patient exposure inherent in these programs may contribute to elevated stress levels. These findings resonate with studies from other educational contexts, emphasizing the need for discipline-specific mental health interventions.

### Risk factors for psychological distress

The multivariable logistic regression analysis confirmed that female gender, early academic year, and enrollment in high-stress academic disciplines were significant predictors of psychological distress. These findings mirror those of previous research, reinforcing the multifactorial nature of mental health risks among healthcare-related students (Nair et al., 2023). Moreover, the consistency of these predictors across different cultural contexts highlights the potential for universally applicable intervention strategies.

### Strengths and limitations

This study’s strengths include its large and diverse sample size, the use of standardized and validated assessment instruments (SCL-90, SDS, SAS), and the comprehensive analysis incorporating multiple demographic and academic variables. However, limitations must be acknowledged. The cross-sectional design restricts causal inferences regarding the temporal relationship between academic factors and mental health outcomes. Reliance on self-reported data may introduce response bias, and the single-center study design may limit the generalizability of the findings to other regions or educational systems.

### Implications for practice and policy

The findings underscore the pressing need for universities to implement targeted mental health interventions. Proactive measures such as early screening, resilience training programs, peer support networks, and access to professional mental health services could mitigate the psychological burden. Particular attention should be directed toward high-risk groups, including female students, early-year cohorts, and students in high-stress academic tracks. Additionally, academic institutions should consider revising curricula to reduce unnecessary academic pressure and promote work-life balance.

### Future research directions

Future research should employ longitudinal designs to elucidate the trajectory of psychological distress across the span of medical education and into early professional practice. Evaluating the effectiveness of targeted intervention strategies through randomized controlled trials will provide robust evidence to inform best practices. Furthermore, exploring the moderating roles of variables such as financial strain, social support, and individual resilience could yield deeper insights into protective factors against psychological distress.

## Conclusion

This study highlights a high prevalence of depressive and anxiety symptoms among Chinese medical university students, with significant variability according to gender, academic year, and discipline. Targeted, evidence-based interventions aimed at enhancing mental health and resilience are urgently needed to safeguard the wellbeing of this vulnerable population and ensure the development of a healthy future healthcare workforce.

## Data Availability

The original contributions presented in this study are included in this article/Supplementary material, further inquiries can be directed to the corresponding authors.
