# Supplementary material for: Mental health and psychological distress in healthcare-related students post-pandemic: a cross-sectional study
Source: Front Psychol. 2026 Jul 20;17:1801247. doi: 10.3389/fpsyg.2026.1801247 (PMC13429662; doi:10.3389/fpsyg.2026.1801247)
Supplement: Supplementary file 1 [file Supplementary_file_1.doc]

Supplementary Table 1. Variable Assignment and Coding Used in Logistic Regression Analyses

| Variable | Coding |
| --- | --- |
| Age | 1.0 = <18 years old; 2.0 = 18–20 years old; 3.0 = >20 years old |
| Gender | 1.0 = Female; 2.0 = Male |
| Department | 1.0 = Traditional Chinese Medicine; 2.0 = Clinical Medicine; 3.0 = Health Management; 4.0 = Medical Technology; 5.0 = Stomatology; 6.0 = Nursing; 7.0 = Pharmacy and Laboratory Medicine |
| Academic Year (Grade) | 2021.0 = Grade 3; 2022.0 = Grade 2; 2023.0 = Grade 1 |
| Major | 1.0 = Traditional Chinese Medicine; 2.0 = Traditional Chinese Medicine Rehabilitation Technology; 3.0 = Chinese Materia Medica; 4.0 = Acupuncture and Tuina; 5.0 = Clinical Medicine; 6.0 = Clinical Medicine (Government-sponsored Program); 7.0 = Health Management; 8.0 = Medical Nutrition; 9.0 = Smart Health and Elderly Care Service and Management; 10.0 = Elderly Health Care and Management; 11.0 = Preventive Medicine; 12.0 = Medical Imaging Technology; 13.0 = Rehabilitation Therapy Technology; 14.0 = Rehabilitation Therapy Technology (International Cooperative Program); 15.0 = Rehabilitation Therapy Technology (University–Enterprise Cooperative Program); 16.0 = Imaging Technology; 17.0 = Optometry Technology; 18.0 = Stomatology; 19.0 = Nursing; 20.0 = Nursing (3+2 Integrated Program); 21.0 = Nursing (International Cooperative Program – Oral Nursing); 22.0 = Nursing (University–Enterprise Cooperative Program – Clinical Research Nursing); 23.0 = Midwifery; 24.0 = Medical Laboratory Technology; 25.0 = Pharmaceutical Production Technology; 26.0 = Pharmaceutical Business and Management; 27.0 = Pharmaceutical Business and Management (University–Enterprise Cooperative Program); 28.0 = Pharmacy |

Supplementary Tables 2 Detection Rates of Depression and Anxiety Among Healthcare-related Students Across Different Dimensions

|  | | Gender（%) | | | |  | | Grade（%) | | | | |
| --- | --- | --- | --- | --- | --- | --- | --- | --- | --- | --- | --- | --- |
|  |  | Male | Female | *χ*2 | *p Value* |  |  | 1 | 2 | 3 | *χ*2 | *p Value* |
| Depression  （*n=*12950） | Asymptomatic | 2411(64.02) | 6329(68.91) | 97.87 | 0.000** |  | Asymptomatic | 3493(65.24) | 2404(63.77) | 2817(74.54) | 163.932 | 0.000** |
|  | Mild | 546(14.50) | 1512(16.46) |  |  | Depression | Mild | 996(18.60) | 581(15.41) | 471(12.46) |  |  |
|  | Moderate | 793(21.06) | 1292(14.07) |  |  | (*n*=12903) | Moderate | 836(15.61) | 761(20.19) | 477(12.62) |  |  |
|  | Severe | 16(0.42) | 51(0.56) |  |  |  | Severe | 29(0.54) | 24(0.64) | 14(0.37) |  |  |
| Anxiety  （*n=*12735） | Asymptomatic | 3332(89.86) | 8121(89.96) | 4.075 | 0.253 |  | Asymptomatic | 4714(88.03) | 3189(88.68) | 3509(93.87) | 93.003 | 0.000** |
|  | Mild | 313(8.44) | 711(7.88) |  |  | Anxiety | Mild | 507(9.47) | 326(9.07) | 187(5.00) |  |  |
|  | Moderate | 47(1.27) | 152(1.68) |  |  | (*n*=12689) | Moderate | 105(1.96) | 60(1.67) | 33(0.88) |  |  |
|  | Severe | 16(0.43) | 43(0.48) |  |  |  | Severe | 29(0.54) | 21(0.58) | 9(0.24) |  |  |

Note: Sample sizes differ between the gender and grade analyses because some participants provided gender, age, and depression/anxiety scale data but did not report their academic year (grade). Consequently, grade-based analyses included only participants with non-missing grade data, whereas gender-based analyses included all participants with available gender information. Therefore, the total across the three grade groups does not equal the full sample used for the gender analyses. *p < 0.05; **p < 0.001.

Supplementary Tables 3 Comparison of SCL-90 Factor Scores Across Academic Departments（n*=*10267）

| **Parameters** | SCL-90 Factor Scores Across Academic Departments **(Mean ± SD)** | | | | | | | **F Value** | ***p* Value** |
| --- | --- | --- | --- | --- | --- | --- | --- | --- | --- |
|  | **TCM** | **Clinical Medicine** | **Health Management** | **Medical Technology** | **Stomatology** | **Nursing** | **Pharmacology** |  |  |
| Somaticatization | 1.28±0.45 | 1.18±0.34 | 1.23±0.39 | 1.20±0.39 | 1.18±0.38 | 1.21±0.42 | 1.24±0.42 | 10.216 | 0.000** |
| Obsessive compulsive Disorder | 1.68±0.70 | 1.54±0.60 | 1.57±0.65 | 1.56±0.60 | 1.45±0.58 | 1.49±0.62 | 1.67±0.67 | 23.599 | 0.000** |
| Interpersonal Sensitivity | 1.49±0.64 | 1.36±0.52 | 1.39±0.56 | 1.39±0.53 | 1.31±0.50 | 1.36±0.55 | 1.47±0.62 | 14.619 | 0.000** |
| Depression | 1.44±0.62 | 1.27±0.44 | 1.35±0.54 | 1.32±0.51 | 1.26±0.46 | 1.31±0.53 | 1.40±0.57 | 17.879 | 0.000** |
| Anxiety | 1.38±0.54 | 1.27±0.42 | 1.31±0.49 | 1.29±0.46 | 1.22±0.41 | 1.27±0.48 | 1.35±0.52 | 13.375 | 0.000** |
| Hostility | 1.36±0.53 | 1.22±0.42 | 1.29±0.50 | 1.25±0.43 | 1.23±0.47 | 1.26±0.48 | 1.31±0.48 | 12.202 | 0.000** |
| Phobic Anxiety | 1.36±0.56 | 1.26±0.45 | 1.30±0.49 | 1.29±0.50 | 1.22±0.43 | 1.28±0.50 | 1.36±0.56 | 10.241 | 0.000** |
| Paranoid Ideation | 1.35±0.53 | 1.24±0.43 | 1.28±0.49 | 1.25±0.43 | 1.22±0.45 | 1.24±0.46 | 1.32±0.51 | 11.785 | 0.000** |
| Psychoticism | 1.32±0.49 | 1.21±0.38 | 1.24±0.43 | 1.24±0.43 | 1.19±0.38 | 1.23±0.44 | 1.29±0.48 | 11.988 | 0.000** |
| Others | 1.38±0.55 | 1.25±0.43 | 1.29±0.47 | 1.28±0.44 | 1.24±0.44 | 1.28±0.48 | 1.33±0.49 | 11.969 | 0.000** |
| Total Score | 125.74±47.46 | 114.48±38.32 | 118.57±42.64 | 116.61±40.64 | 111.20±38.96 | 115.80±42.56 | 123.27±44.44 | 15.505 | 0.000** |
| Mean Score | 1.41±0.51 | 1.29±0.40 | 1.33±0.46 | 1.31±0.43 | 1.25±0.41 | 1.30±0.46 | 1.38±0.48 | 16.128 | 0.000** |

* *p<*0.05 ** *p<*0.001

**Supplementary Tables 4 SDS Scores (Mean ± Standard Deviation) of Healthcare-related Students by Department and Major (n=12,950)**

| **Departments** | **SDS Score**  **（Mean±SD）** | **Major** | **SDS Score**  **（Mean±SD）** |
| --- | --- | --- | --- |
| TCM | 47.22±11.78 | TCM | 46.48±11.75 |
|  |  | Traditional Chinese Rehabilitation Technology | 48.23±11.80 |
|  |  | Chinese Materia Medica | 48.28±12.01 |
|  |  | Acupuncture & Tuina | 46.31±11.51 |
| Clinical Medicine | 42.30±11.53 | Clinical Medicine | 42.41±11.47 |
|  |  | Clinical Medicine (Funded) | 41.98±11.71 |
| Health Management | 45.78±11.57 | Health Management | 45.18±12.71 |
|  |  | Medical Nutrition | 45.93±10.72 |
|  |  | Smart Elderly Care Services and Management | 47.35±11.85 |
|  |  | Elderly Health Care and management | 46.25±11.17 |
|  |  | Preventive Medicine | 44.42±11.36 |
| Medical Technology | 44.19±11.17 | Medical Imaging | 43.04±10.84 |
|  |  | Rehabilitation Therapy Technology | 44.91±11.21 |
|  |  | Rehabilitation Therapy Technology ( Foreign Funded) | 43.52±12.70 |
|  |  | Rehabilitation Therapy Technology ( Local Government Funded) | 40.27±12.10 |
|  |  | Imaging Technology | 46.75±8.02 |
|  |  | Optometry Technology | 45.21±10.80 |
| Stomatology | 45.69±12.34 | Dental Medicine | 45.69±12.34 |
| Nursing | 47.80±11.96 | Nursing | 47.86±12.06 |
|  |  | Nursing（3+2 Integrated Program） | 45.20±10.98 |
|  |  | Nursing（Sino-foreign Joint Dental Nursing Program) | 47.91±11.68 |
|  |  | Nursing（School-Enterprise Cooperative Clinical Research Nurse） | 46.80±12.31 |
|  |  | Mid Wifery | 48.15±11.33 |
| Pharmacology | 47.03±11.23 | Medical Laboratory Technology | 47.80±10.82 |
|  |  | Pharmaceutical Manufacturing technology | 46.50±11.71 |
|  |  | Pharmaceutical business and Management | 43.76±9.72 |
|  |  | Pharmaceutical business and management（Commercial Co-opearation） | 49.12±10.50 |
|  |  | Pharmacy | 47.57±12.34 |
| F Value | 56.647 | F Value | 14.928 |
| *p* Value | 0.000** | *P* Value | 0.000** |

* *p<*0.05 ** *p<*0.001

**Supplementary Tables 5 SAS Scores (Mean ± Standard Deviation) of Healthcare-related Students from Different Departments and Majors (n=12,735)**

| **Department** | **SAS Score**  **（Mean±SD）** | **Major** | **SAS Score**  **（Mean±SD）** |
| --- | --- | --- | --- |
| TCM | 40.34±9.24 | TCM | 40.82±9.76 |
|  |  | Traditional Chinese Rehabilitation Technology | 40.28±8.48 |
|  |  | Chinese Materia Medica | 41.58±9.79 |
|  |  | Acupuncture & Tuina | 38.80±8.61 |
| Clinical Medicine | 37.53±8.56 | Clinical Medicine | 38.05±8.96 |
|  |  | Clinical Medicine (Funded) | 35.99±7.04 |
| Health Management | 39.11±8.79 | Health Management | 38.48±9.09 |
|  |  | Medical Nutrition | 39.02±8.53 |
|  |  | Smart Elderly Care Services and Management | 40.08±8.99 |
|  |  | Elderly Health Care and management | 39.33±8.96 |
|  |  | Preventive Medicine | 38.58±8.40 |
| Medical Technology | 38.06±8.28 | Medical Imaging | 37.64±8.03 |
|  |  | Rehabilitation Therapy Technology | 38.24±8.01 |
|  |  | Rehabilitation Therapy Technology ( Foreign Funded) | 37.20±8.39 |
|  |  | Rehabilitation Therapy Technology ( Local Government Funded) | 34.96±9.27 |
|  |  | Imaging Technology | 41.25±4.50 |
|  |  | Optometry Technology | 38.82±8.65 |
| Stomatology | 38.18±8.44 | Dental Medicine | 38.18±8.44 |
| Nursing | 39.77±8.53 | Nursing | 39.69±8.50 |
|  |  | Nursing（3+2 Integrated Program） | 39.17±8.42 |
|  |  | Nursing（Sino-foreign Joint Dental Nursing Program) | 39.43±8.44 |
|  |  | Nursing（School-Enterprise Cooperative Clinical Research Nurse） | 40.85±9.80 |
|  |  | Mid Wifery | 40.58±8.51 |
| Pharmacology | 39.50±8.63 | Medical Laboratory Technology | 39.74±8.05 |
|  |  | Pharmaceutical Manufacturing technology | 39.06±9.02 |
|  |  | Pharmaceutical business and Management | 37.52±8.18 |
|  |  | Pharmaceutical business and management（Commercial Co-operation） | 39.72±8.70 |
|  |  | Pharmacy | 40.58±9.31 |
| F Value | 25.033 | F Value | 8.371 |
| *p* Value | 0.000** | *p* Value | 0.000** |

* *p<*0.05 ** *p<*0.001
